# Supplementary material for: The Salmonella transmembrane effector SteD hijacks AP1-mediated vesicular trafficking for delivery to antigen-loading MHCII compartments
Source: PLoS Pathog. 2022 May 27;18(5):e1010252. doi: 10.1371/journal.ppat.1010252 (PMC9182567; doi:10.1371/journal.ppat.1010252)
Supplement: S1 Fig — (A) Amino acid sequence of SteD showing regions of amino acids substituted to alanine in alanine scanning mutagenesis. (B) Representative flow cytometry plots showing the gating strategy for GFP-positive cells and negative cells as used for Fig 1C. (C) mMHCII surface levels of Mel JuSo cells expressing GFP or GFP-SteD and treated with DMSO or MG132 were measured by flow cytometry. Mean of three independent experiments done in duplicate ± SD. Data were analysed by paired t-test, ** p<0.01, n.s.–not significant. (PDF) [file ppat.1010252.s001.pdf]

S1 Fig

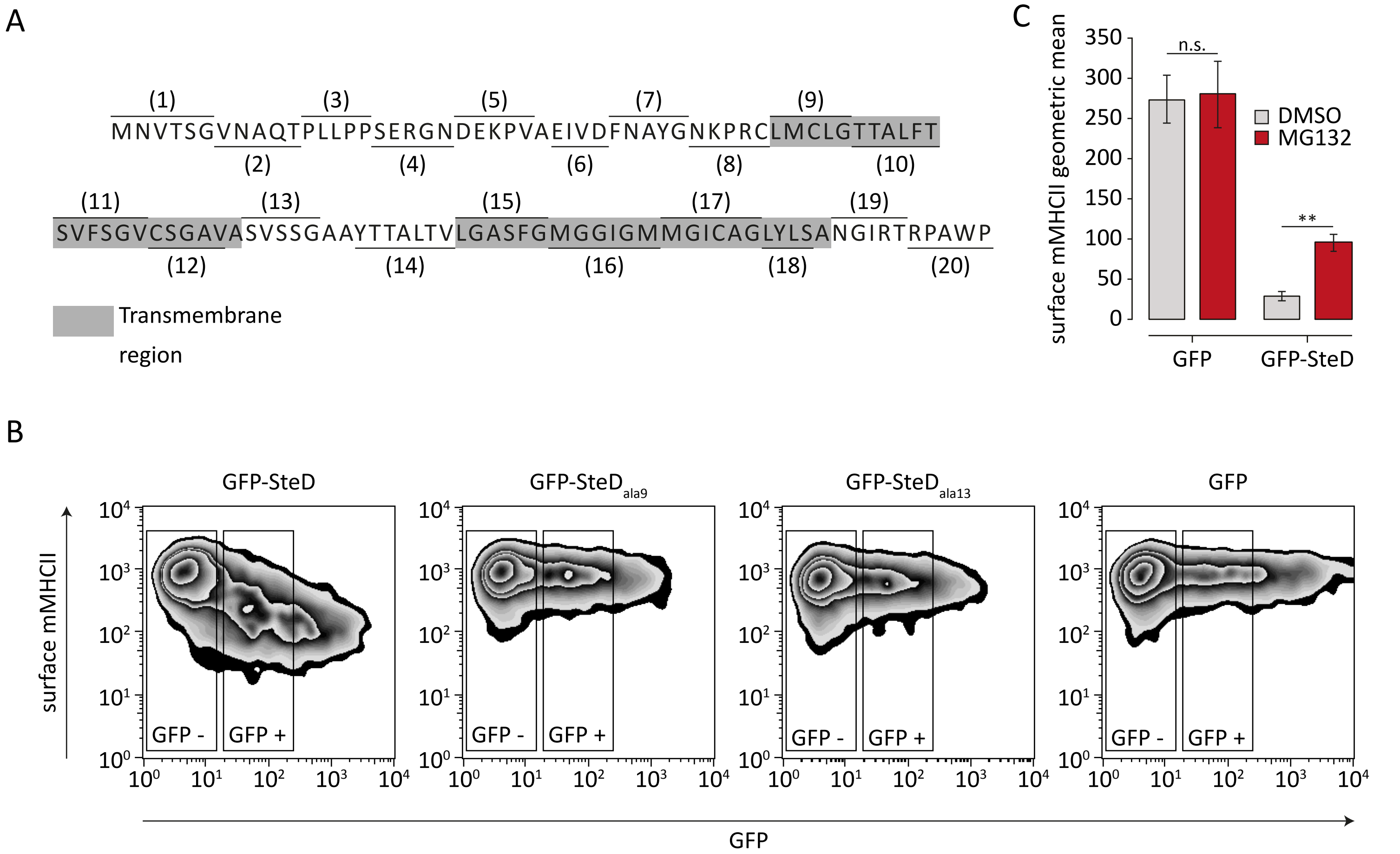

S1 Fig

(A) Amino acid sequence of SteD showing regions of amino acids substituted to alanine in alanine scanning mutagenesis.

(B) Representative flow cytometry plots showing the gating strategy for GFP-positive cells and negative cells as used for Fig 1C.

(C) mMHCII surface levels of Mel JuSo cells expressing GFP or GFP-SteD and treated with DMSO or MG132 were measured by flow cytometry. Mean of three independent experiments done in duplicate ± SD. Data were analysed by paired t-test, ** $p<0.01$, n.s. – not significant.
