# Supplementary material for: The Salmonella transmembrane effector SteD hijacks AP1-mediated vesicular trafficking for delivery to antigen-loading MHCII compartments
Source: PLoS Pathog. 2022 May 27;18(5):e1010252. doi: 10.1371/journal.ppat.1010252 (PMC9182567; doi:10.1371/journal.ppat.1010252)
Supplement: S2 Fig — (A) Representative confocal immunofluorescence microscopy images of Mel JuSo cells expressing GFP-SteD (wt or mutants) after MG132 treatment. Cells were fixed and processed for immunofluorescence microscopy by labelling for ubiquitin (UB, red), and DNA (DAPI, blue). Arrowheads indicate cellular aggregates. Scale bar– 10 μm. (PDF) [file ppat.1010252.s002.pdf]

S2 Fig

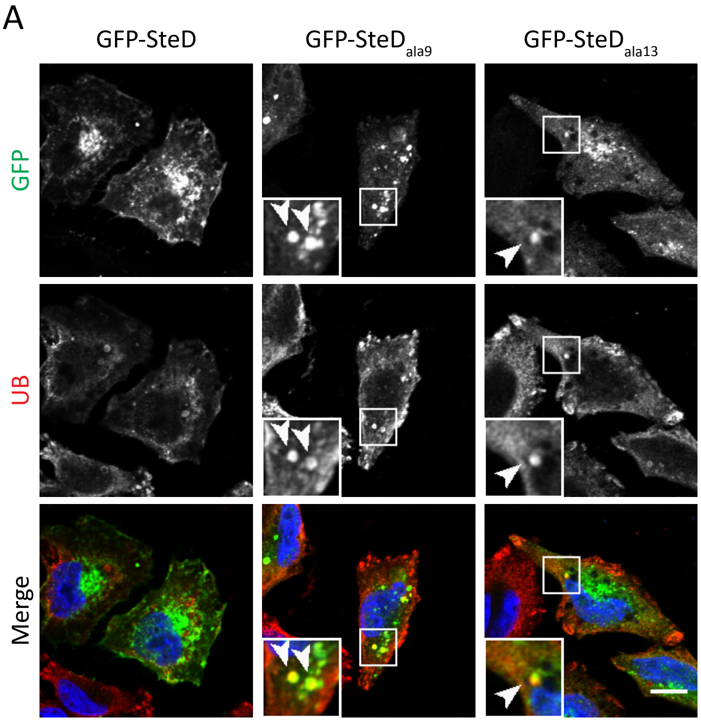

S2 Fig

(A) Representative confocal immunofluorescence microscopy images of Mel JuSo cells expressing GFP-SteD (wt or mutants) after MG132 treatment. Cells were fixed and processed for immunofluorescence microscopy by labelling for ubiquitin (UB, red), and DNA (DAPI, blue). Arrowheads indicate cellular aggregates. Scale bar – 10 µm.
