# Supplementary material for: The Salmonella transmembrane effector SteD hijacks AP1-mediated vesicular trafficking for delivery to antigen-loading MHCII compartments
Source: PLoS Pathog. 2022 May 27;18(5):e1010252. doi: 10.1371/journal.ppat.1010252 (PMC9182567; doi:10.1371/journal.ppat.1010252)
Supplement: S3 Fig — (A and D) Protein immunoblots of whole-cell lysates (WCL) and post-nuclear supernatant (PNS) of Mel Juso cells infected with ΔsteD Salmonella strains carrying a plasmid expressing SteD-HA (wt or mutants) and treated with MG132 or DMSO carrier. Actin and DnaK represent host cell and Salmonella loading controls respectively. (B and C) mMHCII surface levels of Mel JuSo cells expressing GFP or GFP-SteD (wt or mutants) and treated with MG132. Cells were analysed by flow cytometry and amounts of surface mMHCII in GFP-positive cells are expressed as a percentage of GFP-negative cells in the same sample. Mean of three independent experiments done in duplicate ± SD. Data were analysed by one-way ANOVA followed by Dunnett’s multiple comparison test in comparison to wt SteD, *** p<0.001, ** p<0.01, * p<0.05, n.s.–not significant. (E) Representative flow cytometry plots showing the gating strategy for HA-positive and negative cells as used for Fig 3B. (PDF) [file ppat.1010252.s003.pdf]

S3 Fig

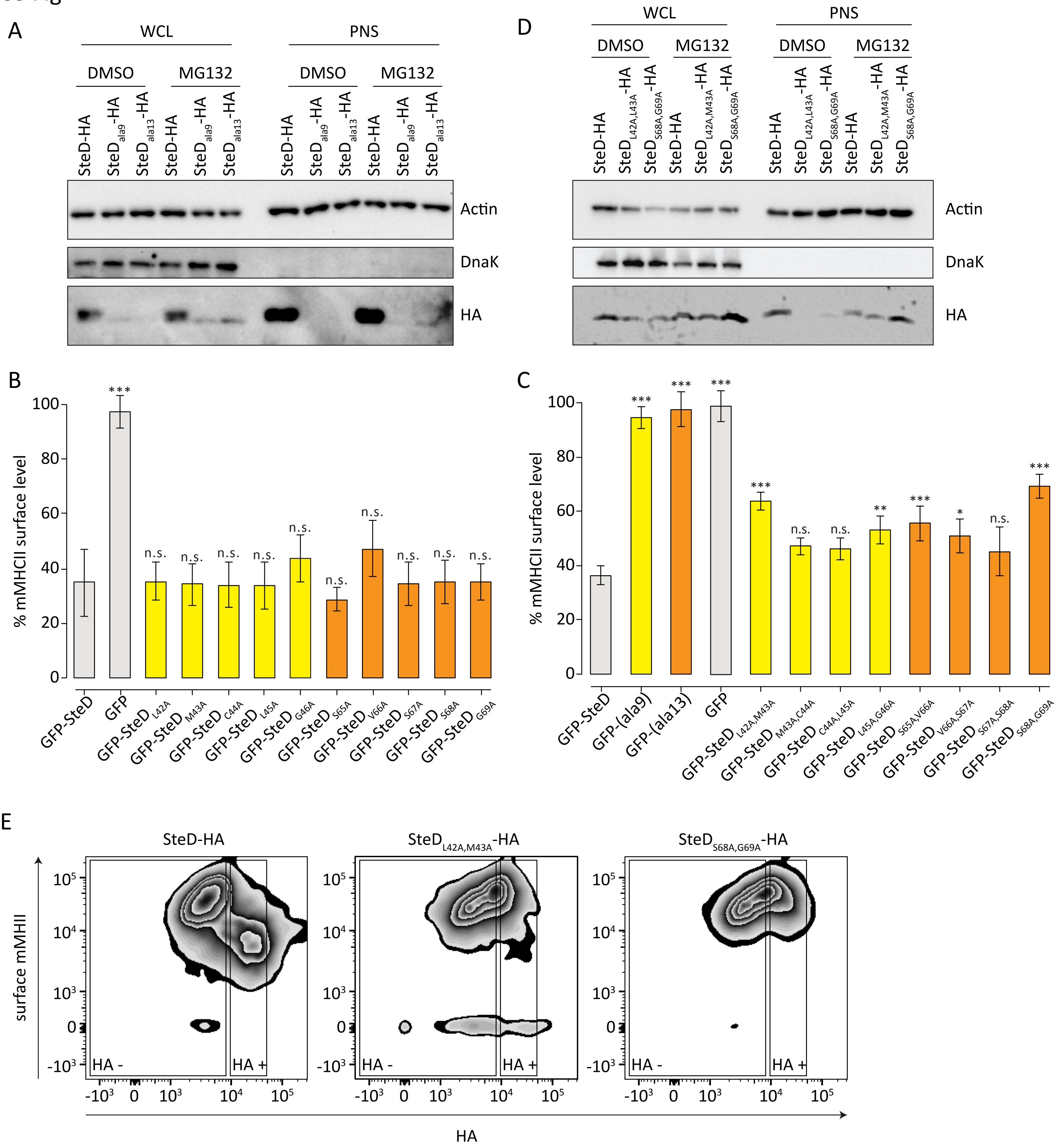

S3 Fig

(A and D) Protein immunoblots of whole-cell lysates (WCL) and post-nuclear supernatant (PNS) of Mel Juso cells infected with *ΔsteD Salmonella* strains carrying a plasmid expressing SteD-HA (wt or mutants) and treated with MG132 or DMSO carrier. Actin and DnaK represent host cell and *Salmonella* loading controls respectively.

(B and C) mMHCII surface levels of Mel JuSo cells expressing GFP or GFP-SteD (wt or mutants) and treated with MG132. Cells were analysed by flow cytometry and amounts of surface mMHCII in GFP-positive cells are expressed as a percentage of GFP-negative cells in the same sample. Mean of three independent experiments done in duplicate ± SD. Data were analysed by one-way ANOVA followed by Dunnett's multiple comparison test in comparison to wt SteD, *** $p<0.001$, ** $p<0.01$, * $p<0.05$, n.s. – not significant.

(E) Representative flow cytometry plots showing the gating strategy for HA-positive and negative cells as used for Fig 3B.
