# Supplementary material for: The Salmonella transmembrane effector SteD hijacks AP1-mediated vesicular trafficking for delivery to antigen-loading MHCII compartments
Source: PLoS Pathog. 2022 May 27;18(5):e1010252. doi: 10.1371/journal.ppat.1010252 (PMC9182567; doi:10.1371/journal.ppat.1010252)
Supplement: S4 Fig — (A) Representative confocal immunofluorescence microscopy images of Mel JuSo cells expressing GFP-SteD under a doxycycline-regulated promoter. Cells were either treated with doxycycline for 4 h (dox) or treated with BFA for 3 h followed by doxycycline and BFA for 4 h (BFA-dox). Cells were then fixed and processed for immunofluorescence microscopy by labelling for MHCII compartments (mMHCII, red), the TGN (TGN46, grey), and DNA (DAPI, blue). Arrowheads indicate MHCII compartments. Scale bar– 10 μm. (B) Representative flow cytometry plots showing the gating strategy for GFP-positive and negative cells as used for Fig 4D. (C) Confocal microscopy images demonstrating photobleaching of a Mel JuSo cell expressing GFP-SteD from S2 Video. Closed arrowheads indicate anterograde vesicle traffic from the Golgi region. Barbed arrowhead indicates retrograde vesicle traffic. Scale bar– 10 μm. (PDF) [file ppat.1010252.s004.pdf]

S4 Fig

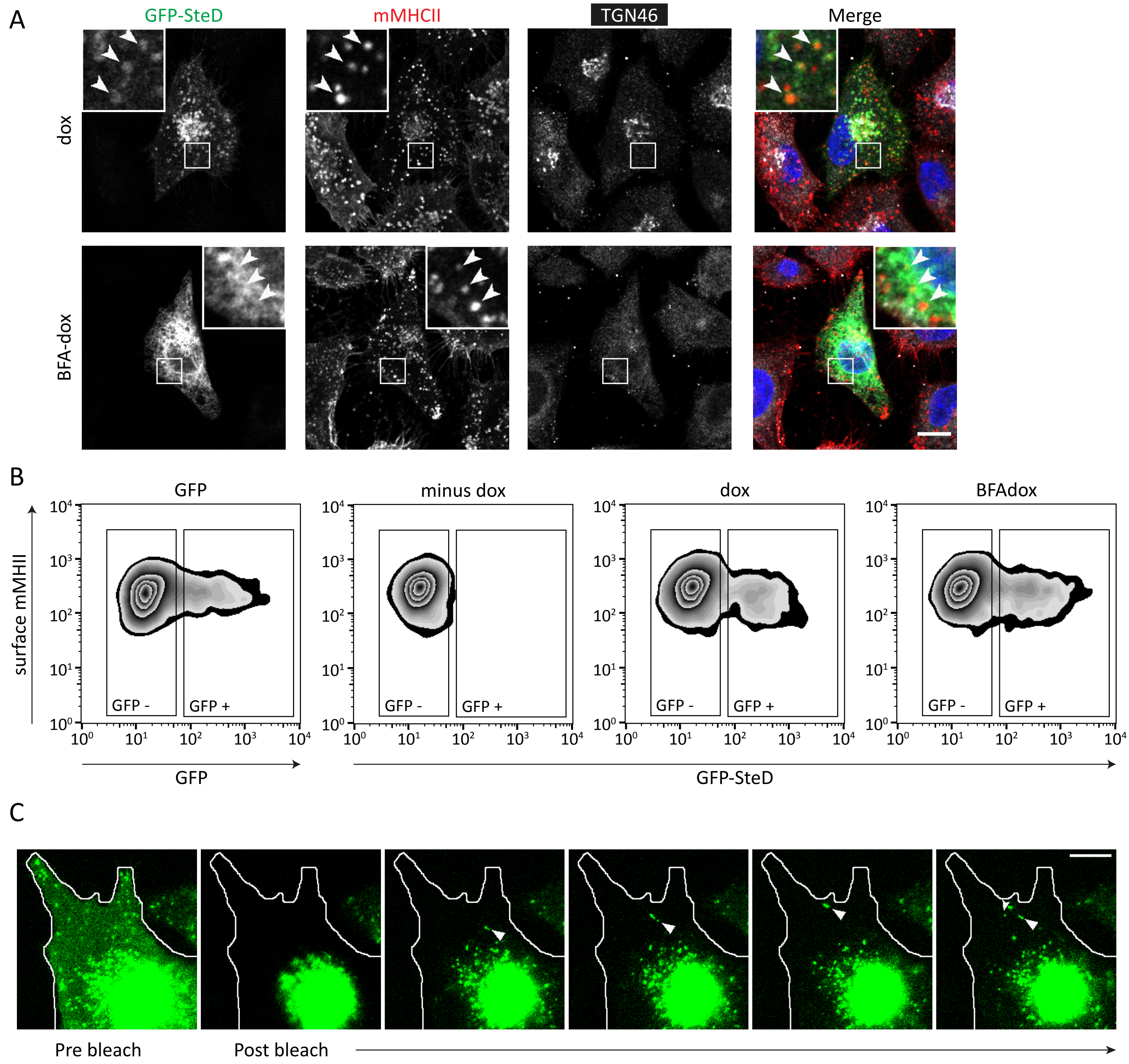

S4 Fig

(A) Representative confocal immunofluorescence microscopy images of Mel JuSo cells expressing GFP-SteD under a doxycycline-regulated promoter. Cells were either treated with doxycycline for 4 h (dox) or treated with BFA for 3 h followed by doxycycline and BFA for 4 h (BFA-dox). Cells were then fixed and processed for immunofluorescence microscopy by labelling for MHCII compartments (mMHCII, red), the TGN (TGN46, grey), and DNA (DAPI, blue). Arrowheads indicate MHCII compartments. Scale bar – 10 μm.

(B) Representative flow cytometry plots showing the gating strategy for GFP-positive and negative cells as used for Fig 4D.

(C) Confocal microscopy images demonstrating photobleaching of a Mel JuSo cell expressing GFP-SteD from S2 Video. Closed arrowheads indicate anterograde vesicle traffic from the Golgi region. Barbed arrowhead indicates retrograde vesicle traffic. Scale bar – 10 μm.
