# Supplementary material for: The Salmonella transmembrane effector SteD hijacks AP1-mediated vesicular trafficking for delivery to antigen-loading MHCII compartments
Source: PLoS Pathog. 2022 May 27;18(5):e1010252. doi: 10.1371/journal.ppat.1010252 (PMC9182567; doi:10.1371/journal.ppat.1010252)
Supplement: S5 Fig — (A) mMHCII surface levels of Mel JuSo cells expressing GFP-SteD and treated with scrambled siRNA (SCR) or siRNA specific to the β subunit of AP1. Cells were analysed by flow cytometry and amounts of surface mMHCII in GFP-positive cells are expressed as a percentage of GFP-negative cells in the same sample. Mean of three independent experiments done in duplicate ± SD. Data were analysed by one-way ANOVA followed by Dunnett’s multiple comparison test n.s.–not significant. (B) Quantification of GFP at the surface of cells represented in Fig 5D. The fluorescence intensity of the GFP signal at the surface of cells was measured in relation to total cellular fluorescence. Data are representative of three independent experiments. Each dot represents the value for one cell. Mean ± SD. The log10 fold change of the data were analysed by t-test, n.s.–not significant. (PDF) [file ppat.1010252.s005.pdf]

S5 Fig

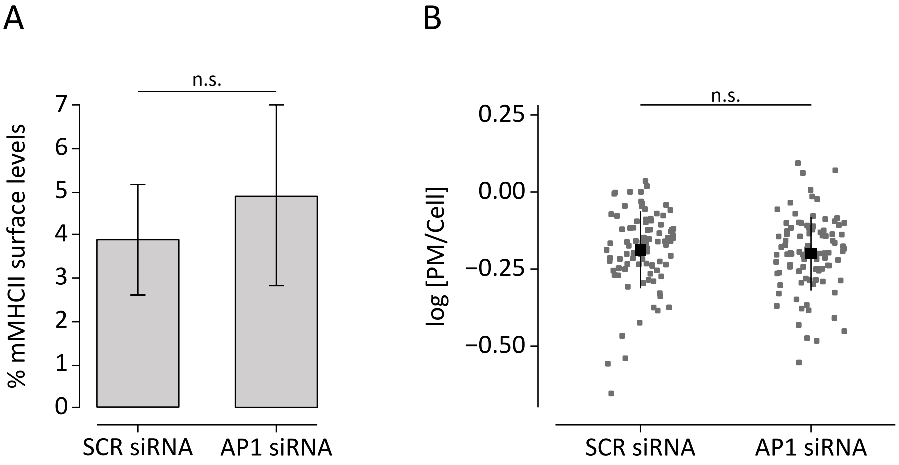

S5 Fig

(A) mMHCII surface levels of Mel JuSo cells expressing GFP-SteD and treated with scrambled siRNA (SCR) or siRNA specific to the β subunit of AP1. Cells were analysed by flow cytometry and amounts of surface mMHCII in GFP-positive cells are expressed as a percentage of GFP-negative cells in the same sample. Mean of three independent experiments done in duplicate ± SD. Data were analysed by one-way ANOVA followed by Dunnett's multiple comparison test n.s. – not significant. (B) Quantification of GFP at the surface of cells represented in Fig 5D. The fluorescence intensity of the GFP signal at the surface of cells was measured in relation to total cellular fluorescence. Data are representative of three independent experiments. Each dot represents the value for one cell. Mean ± SD. The log_{10} fold change of the data were analysed by t-test, n.s. – not significant.
