# Supplementary material for: The Salmonella transmembrane effector SteD hijacks AP1-mediated vesicular trafficking for delivery to antigen-loading MHCII compartments
Source: PLoS Pathog. 2022 May 27;18(5):e1010252. doi: 10.1371/journal.ppat.1010252 (PMC9182567; doi:10.1371/journal.ppat.1010252)
Supplement: S6 Fig — (A) Protein immunoblots of Mel JuSo cells expressing GFP or GFP-SteD (wt or mutants). (B) Quantification of GFP at the TGN of cells represented in Fig 6C. The fluorescence intensity of the GFP signal at the TGN was measured in relation to total cellular fluorescence. Data are representative of three independent experiments. Each dot represents the value for one cell. Mean ± SD. The log10 fold change of the data were analysed by one-way ANOVA followed by Dunnett’s multiple comparison test, *** p<0.001, n.s.–not significant. (C) Confocal microscopy images demonstrating photoactivation of a Mel JuSo cell expressing mEos-SteD (wt or 37–111) from S3 Video. Red dotted circles indicate photo-activated areas. Red arrowheads indicate Golgi-derived vesicles. Scale bar– 10 μm. (PDF) [file ppat.1010252.s006.pdf]

S6 Fig

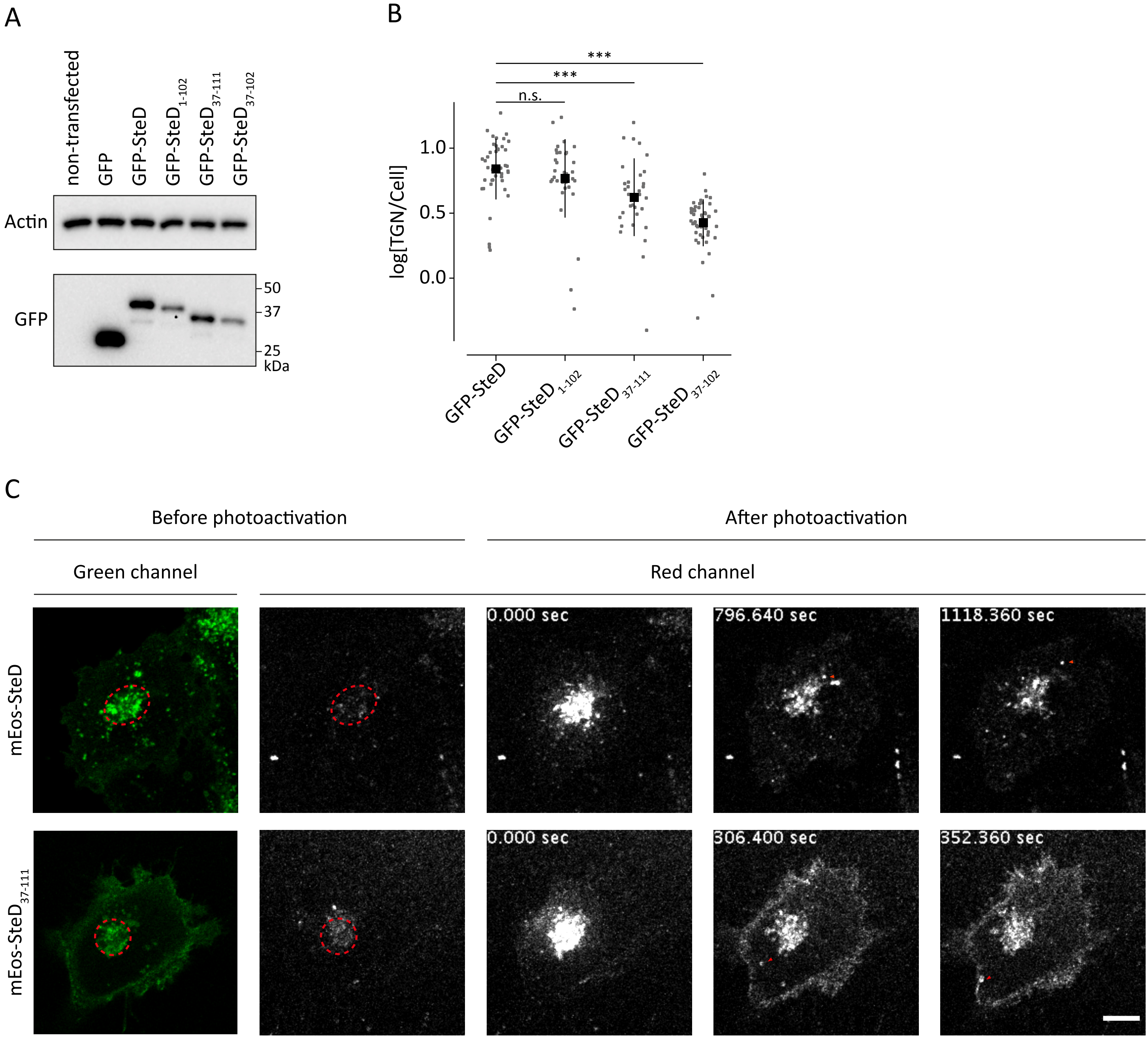

S6 Fig

(A) Protein immunoblots of Mel JuSo cells expressing GFP or GFP-SteD (wt or mutants).

(B) Quantification of GFP at the TGN of cells represented in Fig 6C. The fluorescence intensity of the GFP signal at the TGN was measured in relation to total cellular fluorescence. Data are representative of three independent experiments. Each dot represents the value for one cell. Mean ± SD. The $\log_{10}$ fold change of the data were analysed by one-way ANOVA followed by Dunnett's multiple comparison test, *** p<0.001, n.s. – not significant.

(C) Confocal microscopy images demonstrating photoactivation of a Mel JuSo cell expressing mEos-SteD (wt or 37-111) from S3 Video. Red dotted circles indicate photo-activated areas. Red arrowheads indicate Golgi-derived vesicles. Scale bar – 10 µm.
