# Supplementary material for: The Salmonella transmembrane effector SteD hijacks AP1-mediated vesicular trafficking for delivery to antigen-loading MHCII compartments
Source: PLoS Pathog. 2022 May 27;18(5):e1010252. doi: 10.1371/journal.ppat.1010252 (PMC9182567; doi:10.1371/journal.ppat.1010252)
Supplement: S7 Fig — (A) mMHCII surface levels of Mel JuSo cells expressing GFP or GFP-SteD (wt or mutants). Cells were analysed by flow cytometry and amounts of surface mMHCII in GFP-positive cells are expressed as a percentage of GFP negative cells in the same sample. Mean of three independent experiments done in duplicate ± SD. Data were analysed by one-way ANOVA followed by Dunnett’s multiple comparison test compared to wt SteD, *** p<0.001, ** p<0.01, n.s.–not significant. (B) mMHCII surface levels of Mel Juso cells infected with ΔsteD Salmonella carrying a plasmid expressing SteD-HA (wt or mutant). Cells were analysed by flow cytometry and amounts of surface mMHCII in infected cells are expressed as a percentage of uninfected cells in the same sample. Mean of three independent experiments done in duplicate ± SD. Data were analysed by one-way ANOVA followed by Dunnett’s multiple comparison test, *** p<0.001, n.s.–not significant. (C) Representative confocal immunofluorescence microscopy images of Mel JuSo cells expressing GFP-SteD (wt or mutants). Cells were fixed and processed for immunofluorescence microscopy by labelling for MHCII compartments (mMHCII, red), the TGN (TGN46, grey), and DNA (DAPI, blue). Arrowheads indicate MHCII compartments. Scale bar– 10 μm. (D) Quantification of GFP at the surface of cells represented in S7C Fig. The fluorescence intensity of the GFP signal at the cell surface was measured in relation to total cellular fluorescence. Data are representative of three independent experiments. Each dot represents the value for one cell. Mean ± SD. The log10 fold change of the data were analysed by one-way ANOVA followed by Dunnett’s multiple comparison test, *** p<0.001, n.s.–not significant. (E) Mander’s overlap coefficient of the fraction of GFP-SteD positive pixels that colocalise with mMHCII positive pixels from cells as represented in S7C Fig. Data are representative of three independent experiments. Each dot represents the value for one cell. Mean ± SD. Data [file ppat.1010252.s007.pdf]

S7 Fig

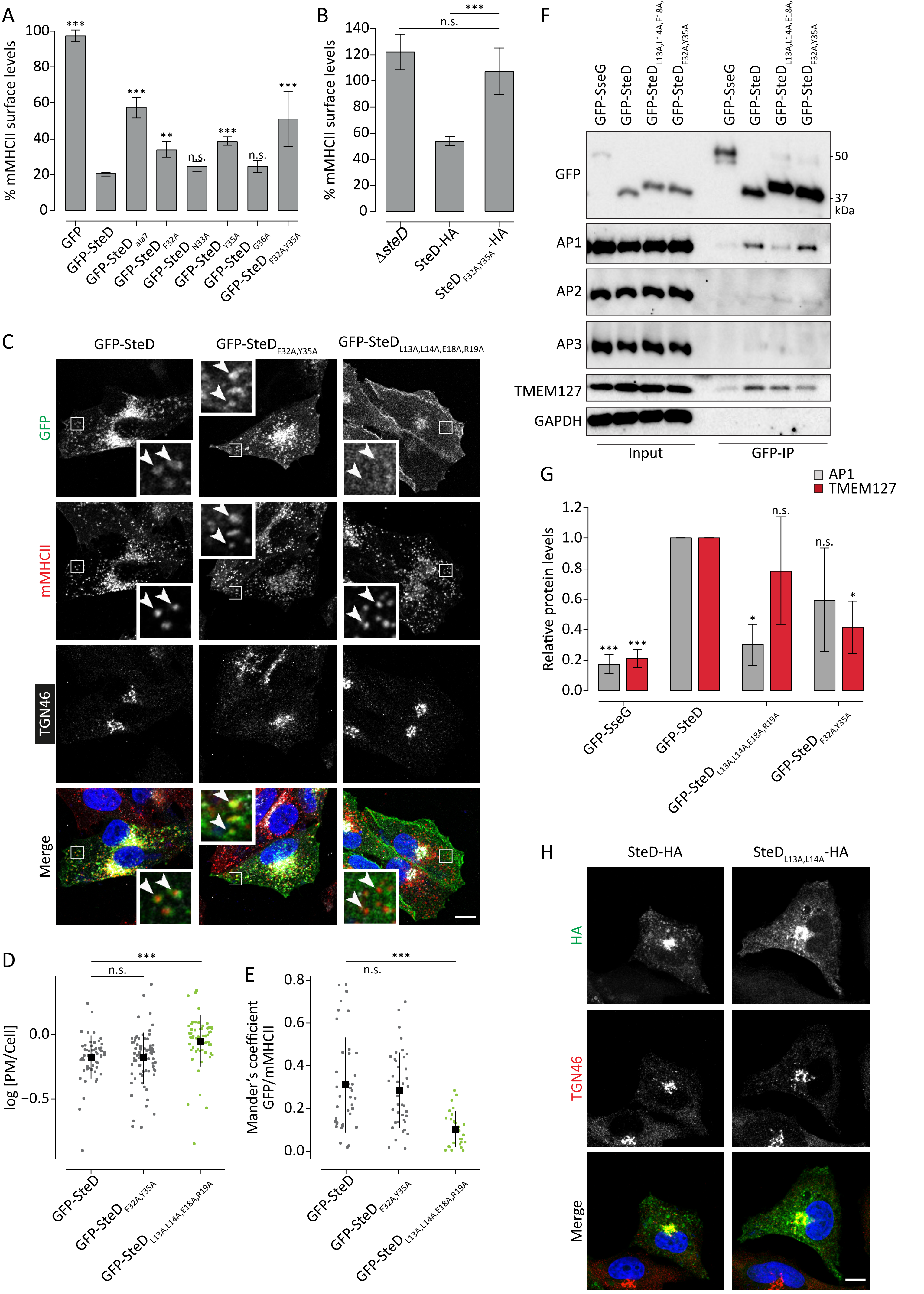
A
% mMHCII surface levels
GFP
GFP-SteD
GFP-SteD ala7
GFP-SteD F32A
GFP-SteD N33A
GFP-SteD Y35A
GFP-SteD G36A
GFP-SteD F32A,Y35A
B
ΔsteD
SteD-HA
SteD F32A,Y35A -HA
C
GFP-SteD
GFP-SteD F32A,Y35A
GFP-SteD L13A,L14A,E18A,R19A
GFP
mMHCII
TGN46
Merge
D
log [PM/Cell]
E
Mander's coefficient GFP/mMHCII
F
GFP-SseG
GFP-SteD
GFP-SteD L13A,L14A,E18A,R19A
GFP-SteD F32A,Y35A
GFP
AP1
AP2
AP3
TMEM127
GAPDH
Input
GFP-IP
50
37
kDa
G
AP1
TMEM127
Relative protein levels
H
SteD-HA
SteD L13A,L14A -HA
HA
TGN46
Merge

S7 Fig

(A) mMHCII surface levels of Mel JuSo cells expressing GFP or GFP-SteD (wt or mutants). Cells were analysed by flow cytometry and amounts of surface mMHCII in GFP-positive cells are expressed as a percentage of GFP negative cells in the same sample. Mean of three independent experiments done in duplicate ± SD. Data were analysed by one-way ANOVA followed by Dunnett's multiple comparison test compared to wt SteD, *** p<0.001, ** p<0.01, n.s. – not significant.

(B) mMHCII surface levels of Mel Juso cells infected with *ΔsteD Salmonella* carrying a plasmid expressing SteD-HA (wt or mutant). Cells were analysed by flow cytometry and amounts of surface mMHCII in infected cells are expressed as a percentage of uninfected cells in the same sample. Mean of three independent experiments done in duplicate ± SD. Data were analysed by one-way ANOVA followed by Dunnett's multiple comparison test, *** p<0.001, n.s. – not significant.

(C) Representative confocal immunofluorescence microscopy images of Mel JuSo cells expressing GFP-SteD (wt or mutants). Cells were fixed and processed for immunofluorescence microscopy by labelling for MHCII compartments (mMHCII, red), the TGN (TGN46, grey), and DNA (DAPI, blue). Arrowheads indicate MHCII compartments. Scale bar – 10 µm.

(D) Quantification of GFP at the surface of cells represented in S7C Fig. The fluorescence intensity of the GFP signal at the cell surface was measured in relation to total cellular fluorescence. Data are representative of three independent experiments. Each dot represents the value for one cell. Mean ± SD. The $\log_{10}$ fold change of the data were analysed by one-way ANOVA followed by Dunnett's multiple comparison test, *** p<0.001, n.s. – not significant.

(E) Mander's overlap coefficient of the fraction of GFP-SteD positive pixels that colocalise with mMHCII positive pixels from cells as represented in S7C Fig. Data are representative of three independent experiments. Each dot represents the value for one cell. Mean ± SD. Data were analysed by one-way ANOVA followed by Dunnett's multiple comparison test, *** p<0.001, n.s. – not significant.

(F) Protein immunoblots of whole-cell lysates (Input) and immunoprecipitation with GFP-trap beads (GFP IP) from Mel Juso cells expressing GFP-SteD (wt or mutants) or GFP-SseG following crosslinking with DSP. Mutation of charged residues might explain the difference in migration through the SDS gel. AP1 – antibody specific for the γ subunit, AP2 – antibody specific for the α subunit, AP3 – antibody specific for the δ subunit.

(G) Levels of immunoprecipitated AP1 and TMEM127 were calculated by densitometry from immunoblots as represented in S7F Fig. Protein levels were normalised to GFP-SteD. Mean of three independent experiments ± SD. The data were analysed by one sample t-test, *** p<0.001, * p<0.05, n.s. – not significant.

(H) Representative confocal immunofluorescence microscopy images of Mel Juso cells infected with *ΔsteD Salmonella* strains carrying a plasmid expressing SteD-HA (wt or mutant). Cells were fixed and processed for immunofluorescence microscopy by labelling for HA (green), the TGN (TGN46, red), and DNA (DAPI, blue). Scale bar – 10 µm.
