# Supplementary material for: The Salmonella transmembrane effector SteD hijacks AP1-mediated vesicular trafficking for delivery to antigen-loading MHCII compartments
Source: PLoS Pathog. 2022 May 27;18(5):e1010252. doi: 10.1371/journal.ppat.1010252 (PMC9182567; doi:10.1371/journal.ppat.1010252)
Supplement: S1 Table — (PDF) [file ppat.1010252.s011.pdf]

S1 Table. ***S.*** **Typhimurium strains used in this study**

Name	Description	Reference
wild-type	14028s *S.* Typhimurium wild-type	ATCC
ΔssaV	*ΔssaV* ::km	[1]
ΔsteD	*ΔsteD* ::km	[2]

Abbreviations: ATCC - American Type Culture Collection, km - kanamycin resistance

References

1. Beuzón CR, Banks G, Deiwick J, Hensel M, Holden DW. pH-dependent secretion of SseB, a product of the SPI-2 type III secretion system of Salmonella typhimurium. Mol Microbiol. 1999;33: 806–16. doi:10.1046/j.1365-2958.1999.01527.x

2. Bayer-Santos E, Durkin CH, Rigano LA, Kupz A, Alix E, Cerny O, et al. The Salmonella Effector SteD Mediates MARCH8-Dependent Ubiquitination of MHC II Molecules and Inhibits T Cell Activation. Cell Host Microbe. 2016;20: 584–595. doi:10.1016/j.chom.2016.10.007
