# Supplementary material for: The Salmonella transmembrane effector SteD hijacks AP1-mediated vesicular trafficking for delivery to antigen-loading MHCII compartments
Source: PLoS Pathog. 2022 May 27;18(5):e1010252. doi: 10.1371/journal.ppat.1010252 (PMC9182567; doi:10.1371/journal.ppat.1010252)
Supplement: S2 Table — (PDF) [file ppat.1010252.s012.pdf]

S2 Table. Plasmids used in this study

Name	Description	Reference
m4p GFP	Mammalian retroviral expression plasmid containing GFP	[1]
m4p GFP-SteD	Mammalian retroviral expression plasmid containing N-terminal GFP-tagged steD	[1]
m4p GFP-SteD$_{ala9}$	Mammalian retroviral expression plasmid containing N-terminal GFP-tagged steD$_{ala9}$	[1]
m4p GFP-SteD$_{ala13}$	Mammalian retroviral expression plasmid containing N-terminal GFP-tagged steD$_{ala13}$	[1]
m4p mCherry-SseG	Mammalian retroviral expression plasmid containing N-terminal mCherry-tagged sseG	This study
m4p GFP-SteD(SseG TM)	Mammalian retroviral expression plasmid containing N-terminal GFP-tagged chimera of steD with TM domains of sseG	This study
m4p GFP-SteD(SseG TM)$_{ala13}$	Mammalian retroviral expression plasmid containing N-terminal GFP-tagged chimera of steD$_{ala13}$ with TM domains of sseG	This study
m4p GFP-SteD$_{LM42AA}$	Mammalian retroviral expression plasmid containing N-terminal GFP-tagged steD$_{LM42AA}$	This study
m4p GFP-SteD$_{MC43AA}$	Mammalian retroviral expression plasmid containing N-terminal GFP-tagged steD$_{MC43AA}$	This study
m4p GFP-SteD$_{CL44AA}$	Mammalian retroviral expression plasmid containing N-terminal GFP-tagged steD$_{CL44AA}$	This study
m4p GFP-SteD$_{LG45AA}$	Mammalian retroviral expression plasmid containing N-terminal GFP-tagged steD$_{LG45AA}$	This study
m4p GFP-SteD$_{SV65AA}$	Mammalian retroviral expression plasmid containing N-terminal GFP-tagged steD$_{SV65AA}$	This study
m4p GFP-SteD$_{VS66AA}$	Mammalian retroviral expression plasmid containing N-terminal GFP-tagged steD$_{VS66AA}$	This study

m4p GFP-$SteD_{SS67AA}$	Mammalian retroviral expression plasmid containing N-terminal GFP-tagged $steD_{SS67AA}$	This study
m4p GFP-$SteD_{SG68AA}$	Mammalian retroviral expression plasmid containing N-terminal GFP-tagged $steD_{SG68AA}$	This study
pWSK29 pSteD-SteD-2HA,SrcA	*Salmonella* expression plasmid containing C-terminal 2HA-tagged steD and srcA including intergenic region with endogenous promoter	[2]
pWSK29 pSteD-$SteD_{LM42AA}$-2HA,SrcA	*Salmonella* expression plasmid containing C-terminal 2HA-tagged $steD_{LM42AA}$ and srcA including intergenic region with endogenous promoter	This study
pWSK29 pSteD-$SteD_{SG68AA}$-2HA,SrcA	*Salmonella* expression plasmid containing C-terminal 2HA-tagged $steD_{SG68AA}$ and srcA including intergenic region with endogenous promoter	This study
pcDNA 6/TR	Mammalian expression plasmid for high-level expression of the tetracycline repressor (TR) protein	Life Technologies
pcDNA 4/TO GFP-SteD	Mammalian expression plasmid for doxycycline-regulated expression of N-terminal tagged GFP-steD	This study
pcDNA 4/TOGFP-$SteD_{ala13}$	Mammalian expression plasmid for doxycycline-regulated expression of N-terminal tagged GFP-$steD_{ala13}$	This study
m4p GFP-$SteD_{(1-102)}$	Mammalian retroviral expression plasmid containing N-terminal GFP-tagged steD(1-102)	This study
m4p GFP-$SteD_{(37-111)}$	Mammalian retroviral expression plasmid containing N-terminal GFP-tagged steD(37-111)	This study
m4p GFP-$SteD_{(37-102)}$	Mammalian retroviral expression plasmid containing N-terminal GFP-tagged steD(37-102)	This study
m4p mEos3.2-SteD	Mammalian retroviral expression plasmid containing N-terminal mEos3.2-tagged steD	This study with mEos3.2 from Addgene 57484
m4p mEos3.2-$SteD_{(37-111)}$	Mammalian retroviral expression plasmid containing N-terminal mEos3.2-tagged steD(37-111)	This study with mEos3.2 from Addgene 57484
pWSK29 pSteD-$SteD_{LL13AA}$,SrcA	*Salmonella* expression plasmid containing C-terminal 2HA-tagged $steD_{LL13AA}$ and srcA including intergenic region with endogenous promoter	This study

pWSK29 pSteD-SteD$_{LL/ER}$-2HA,SrcA	*Salmonella* expression plasmid containing C-terminal 2HA-tagged steD$_{LL/ER}$ and srcA including intergenic region with endogenous promoter	This study
pWSK29 pSseA-SseF-2HA	*Salmonella* expression plasmid containing C-terminal 2HA-tagged sseF with sseA promoter	[3]
m4p GFP-SteD$_{LL/ER}$	Mammalian retroviral expression plasmid containing N-terminal GFP-tagged steD$_{LL/ER}$	This study
mCherry-ER-3	Mammalian expression plasmid for expression of	Gift from Michael Davidson (Addgene 55041)

References

1. Bayer-Santos E, Durkin CH, Rigano LA, Kupz A, Alix E, Cerny O, et al. The Salmonella Effector SteD Mediates MARCH8-Dependent Ubiquitination of MHC II Molecules and Inhibits T Cell Activation. Cell Host Microbe. 2016;20: 584–595. doi:10.1016/j.chom.2016.10.007

2. Godlee C, Cerny O, Durkin CH, Holden DW. SrcA is a chaperone for the Salmonella SPI-2 type three secretion system effector SteD. Microbiology. 2019;165: 15–25. doi:10.1099/mic.0.000732

3. Yu X-J, McGourty K, Liu M, Unsworth KE, Holden DW. pH sensing by intracellular Salmonella induces effector translocation. Science. 2010;328: 1040–3. doi:10.1126/science.1189000
