# Supplementary material for: The Salmonella transmembrane effector SteD hijacks AP1-mediated vesicular trafficking for delivery to antigen-loading MHCII compartments
Source: PLoS Pathog. 2022 May 27;18(5):e1010252. doi: 10.1371/journal.ppat.1010252 (PMC9182567; doi:10.1371/journal.ppat.1010252)
Supplement: S3 Table — (PDF) [file ppat.1010252.s013.pdf]

S3. Primary antibodies used in this study

Antibody	Source	Use	Dilution
Rat monoclonal anti-GFP clone 3H9	Chromotek 3H9-100	WB	1 in 2000
Rabbit anti-actin	Sigma A2066	WB	1 in 2000
Mouse monoclonal HRP-conjugated anti-ubiquitin clone P4D1	Santa Cruz sc-8017	WB	1 in 500
Mouse monoclonal anti-Golgin97 clone CDF4	eBioscience 14-9767-82	WB	1 in 1000
Mouse monoclonal anti-HLA-DR alpha chain clone TAL.1B5	DAKO M0746	WB	1 in 3000
Mouse monoclonal anti-HLA-DR cloneL243	Sigma-Aldrich	Flow/IF	Flow and IF 1 in 300
Rabbit anti-TGN46	LSBio LS-B6874	IF	1 in 400
Mouse monoclonal anti-ubiquitin clone FK2	Enzo BML-PW8810	IF	1 in 100
Mouse monoclonal anti-DnaK clone 8E2/2	Enzo ADI-SPA-880-F	WB	1 in 2000
Mouse monoclonal anti-HA 16B12	Biolegend 901502	WB	1 in 1000
Rat monoclonal anti-HA clone 3F10	Roche 11867423001	IF	1 in 200
Rabbit anti-beta-1 adaptin	Thermo Fisher PA5-66994	WB	1 in 500
Mouse anti-gamma adaptin clone 100/3	Sigma A4200	WB	1 in 100
Mouse anti-adaptin alpha clone 8	BD biosciences 610502	WB	1 in 1000

Mouse anti-adaptin delta clone 18	BD Biosciences 611329	WB	1 in 1000
Rabbit anti-GAPDH	Abcam ab9585	WB	1 in 1000
Rabbit anti-TMEM127	Bethyl Laboratories A303-450A	WB	1 in 500

Abbreviations: IF - immunofluorescence, WB - western blot
