# Supplementary material for: The Salmonella transmembrane effector SteD hijacks AP1-mediated vesicular trafficking for delivery to antigen-loading MHCII compartments
Source: PLoS Pathog. 2022 May 27;18(5):e1010252. doi: 10.1371/journal.ppat.1010252 (PMC9182567; doi:10.1371/journal.ppat.1010252)
Supplement: S4 Table — (PDF) [file ppat.1010252.s014.pdf]

**S4 Table. Primers used in this study**

| Sequence 5'-3' | Description | Plasmid/s created | |
|---|---|---|---|
| TAAGCAACATGTCAatgaaacctgttagcccaaatg | Forward primer for SseG with PciI | m4p mCherry-SseG | |
| ATCGATGCGGCCGCTTActccggcg | Reverse primer for SseG with NotI | m4p mCherry-SseG | |
| TAAGCAACATGTCAatgaatgtcacttcaggcg | Primer A for SteD with PciI | m4p GFP-SteD | |
| ATCGATGCGGCCGCTTATGGCCAGGCTG | Primer B for SteD with NotI | m4p GFP-SteD | |
| TAAGCAAAGCTTGATATCGAATTCtgataacgaaacggag | Primer A for SteD-2HA with HindIII | pWSK29 pSteD-SteD-2HA, SrcA | |
| ATCGATGCGGCCGCttagaggctag | Primer B for SteD-2HA with NotI | pWSK29 pSteD-SteD-2HA, SrcA | |
| CCGCGATGGCAATAGCAAGGCCAATCCCTGCCAGGGCTACaaccgttagagcggtagtatatg | Primer B for overlap PCR | m4p GFP-SteD(SseG TM) | |
| CCTTGCTATTGCCATCGCGGATGTCGCCTGTCTTATCTacaatggcatcagaacccgg | Primer C for overlap PCR | m4p GFP-SteD(SseG TM) | |
| GCATAAAATCTGTCCGCCCAAAATAACCGTTTGCCAACTAAAAATtctgggtttattgccgtaagcgttaaaatc | Primer B for overlap PCR | m4p GFP-SteD(SseG TM) | |
| TTGGGCGGACAGATTTTATGCTGTTCCGCTGGAATAGCATTAACAagtgtcagtagtggtgcggcatatac | Primer C for overlap PCR | m4p GFP-SteD(SseG TM) | |
| agtataAGCTGCAGCTGCAGCTGCAGCTGTTAATGCTATTCCAGCGGAAC | Primer B for overlap PCR | m4p GFP-SteD(SseG TM)$_{ala13}$ | |
| TTAACAGCTGCAGCTGCAGCTGCAGCTtatactaccgctctaacggttG | Primer C for overlap PCR | m4p GFP-SteD(SseG TM)$_{ala13}$ | |
| ggttcccaggcaAGCTGCacatctgggtttattg | Primer B for overlap PCR | m4p GFP-SteD$_{LM42AA}$ | pWSK29 pSteD-SteD$_{LM42AA}$-2HA,SrcA |

| | | | |
|---|---|---|---|
| caataaacccagatgtGCAGCTtgcctgggaacc | Primer C for overlap PCR | m4p GFP-SteD$_{LM42AA}$ | pWSK29 pSteD-SteD$_{LM42AA}$-2HA,SrcA |
| cgtggttcccagAGCTGCtaaacatctgggtttattg | Primer B for overlap PCR | m4p GFP-SteD$_{MC43AA}$ | |
| caataaacccagatgtttaGCAGCTctgggaaccacg | Primer C for overlap PCR | m4p GFP-SteD$_{MC43AA}$ | |
| gccgtggttccAGCTGCcattaaacatctggg | Primer B for overlap PCR | m4p GFP-SteD$_{CL44AA}$ | |
| cccagatgtttaatgGCAGCTggaaccacggc | Primer C for overlap PCR | m4p GFP-SteD$_{CL44AA}$ | |
| caatgccgtggtAGCTGCgcacattaaacatctg | Primer B for overlap PCR | m4p GFP-SteD$_{LG45AA}$ | |
| cagatgtttaatgtgcGCAGCTaccacggcattg | Primer C for overlap PCR | m4p GFP-SteD$_{LG45AA}$ | |
| cgcaccactactAGCTGCggcgacagccc | Primer B for overlap PCR | m4p GFP-SteD$_{SV65AA}$ | |
| gggctgtcgccGCAGCTagtagtggtgcg | Primer C for overlap PCR | m4p GFP-SteD$_{SV65AA}$ | |
| gccgcaccactAGCTGCactggcgacagc | Primer B for overlap PCR | m4p GFP-SteD$_{VS66AA}$ | |
| gctgtcgccagtGCAGCTagtggtgcggc | Primer C for overlap PCR | m4p GFP-SteD$_{VS66AA}$ | |
| gtatatgccgcaccAGCTGCgacactggcgacag | Primer B for overlap PCR | m4p GFP-SteD$_{SS67AA}$ | |
| ctgtcgccagtgtcGCAGCTggtgcggcatatac | Primer C for overlap PCR | m4p GFP-SteD$_{SS67AA}$ | |
| gtagtatatgccgcAGCTGCactgacactggcg | Primer B for overlap PCR | m4p GFP-SteD$_{SG68AA}$ | pWSK29 pSteD-SteD$_{SG68AA}$-2HA,SrcA |

| | | | |
|---|---|---|---|
| cgccagtgtcagtGCAGCTgcggcatatactac | Primer C for overlap PCR | m4p GFP-SteD$_{SG68AA}$ | pWSK29 pSteD-SteD$_{SG68AA}$-2HA,SrcA |
| GGATCCGGATCCATGGTGAGCAAGGGCGAGGAG | Forward primer for GFP with BamHI | pcDNA 4/TO GFP-SteD | pcDNA 4/TOGFP-SteD$_{ala13}$ |
| ATCGATGAATTCttatggccaggctggccgg | Reverse primer for SteD with EcoRI | pcDNA 4/TO GFP-SteD | pcDNA 4/TOGFP-SteD$_{ala13}$ |
| TAAGCAACATGTCAaataaacccagatgtttaatgtg | Forward primer for SteD truncation from residue 37 with Pci1 | m4p GFP-SteD$_{(37-111)}$ | m4p GFP-SteD$_{(37-102)}$ |
| ATCGATGCGGCCGCTTAatttgcgctaaggtatagtc | Reverse primer for SteD truncation to residue 102 with Not1 | m4p GFP-SteD$_{(1-102)}$ | m4p GFP-SteD$_{(37-102)}$ |
| TAAGCACCATGGTGagtgcgattaagccagacatg | Forward primer for mEos3.2 with Nco1 | m4p mEos3.2-SteD | m4p mEos3.2-SteD$_{(37-111)}$ |
| ATCGATACATGTCtcgtctggcattgtcaggc | Reverse primer for mEos3.2 with Pci1 | m4p mEos3.2-SteD | m4p mEos3.2-SteD$_{(37-111)}$ |
| CATTACCCCGTTCAGAAGGAGGGGCCGCTGGCGTTTGCGCATTCAC | Primer B for overlap PCR | pWSK29 pSteD-SteD$_{LL13AA}$,SrcA | |
| GTGAATGCGCAAACGCCAGCGGCCCCTCCTTCTGAACGGGGTAATG | Primer C for overlap PCR | pWSK29 pSteD-SteD$_{LL13AA}$,SrcA | |
| ttaccAGCTGCagaaggaggAGCTGCtggcgtttgcgcattcacg | Primer B for overlap PCR | pWSK29 pSteD-SteD$_{LL/ER}$-2HA,SrcA | m4p GFP-SteD$_{LL/ER}$ |
| acgccaGCAGCTcctccttctGCAGCTggtaatgacgaaaaaccggtagctg | Primer C for overlap PCR | pWSK29 pSteD-SteD$_{LL/ER}$-2HA,SrcA | m4p GFP-SteD$_{LL/ER}$ |
