# Supplementary material for: The Salmonella transmembrane effector SteD hijacks AP1-mediated vesicular trafficking for delivery to antigen-loading MHCII compartments
Source: PLoS Pathog. 2022 May 27;18(5):e1010252. doi: 10.1371/journal.ppat.1010252 (PMC9182567; doi:10.1371/journal.ppat.1010252)
Supplement: Attachment — Submitted filename: Godlee2022_AuthorResponse.docx [file ppat.1010252.s015.docx]

Dear Editor,

Many thanks for the referees’ comments and for giving us the opportunity to send you a revised manuscript. Below we highlight the criticisms of the referees and provide our responses:

**Major Issues**

Reviewer #1:

*None*

Reviewer #2:   
*In part I, the authors analyze the membrane integration of alanine mutants of SteD. They find that mutation of sequences in the beginning of as well as right behind transmembrane segment 1 (TMS1) result in destabilized, non-functional proteins that cannot be extracted from a membrane fraction, neither with 2.5 M urea nor with RIPA buffer. Surprisingly, the authors conclude that both mutants fail to integrate into membranes. Neither does failed extraction with a low concentration of 2.5 M urea prove membrane integration nor does failed extraction with RIPA buffer disprove membrane integration. If these mutants are truly not membrane integrated, the authors should show this by extracting with 8 M urea instead. Specific interactions of peripheral membrane proteins may be disrupted with 2.5 M urea (as shown for Golgin-97) but aggregates may not be solubilized at this low concentration of urea but at a higher one. Also, intramembrane aggregates exist that resist extraction with fairly mild buffers like RIPA. Typically, intramembrane aggregates are revealed by their extractability using 1% Fos-Choline 12, which could be used by the authors.*

**Response**

The reviewer points out that a lack of extraction by 2.5 M urea does not prove membrane integration. This is not something that we claim. We state that urea is used “to extract peripheral membrane proteins, including Golgin-97” on lines 86-87. We have removed from the methods the indication that urea wash yielded “a pellet containing integral membrane proteins” on line 437 as this could be misleading.

The reviewer suggests that we test for extraction of both mutants with 8 M urea, which can solubilise aggregates. We have carried out this experiment and indeed both SteD_ala9_ and SteD_ala13_ were extracted by 8 M urea (Fig R1A, below). However, wt SteD and the integral membrane control DRα were also extracted by 8 M urea (Fig R1A). This is consistent with 8 M urea being a strong denaturant. As 8 M urea did not distinguish between cytosolic aggregates and integral membrane proteins, we do not think that the result of the experiment adds to the manuscript.

The reviewer is concerned that the SteD_ala13_ mutant might form aggregates within the membrane and suggests that the alternative detergent fos-choline 12 could be used to extract integral membrane aggregates. Despite extensive searches, we could not find any examples of this detergent being used in a biochemical assay to distinguish between integral membrane aggregates and cytoplasmic aggregates. Indeed, it is used predominantly to extract membrane proteins for structural and biochemical studies. It has been shown that fos-choline 12 is very efficient at solubilising misfolded membrane proteins (PMID: 25454020). We found that fos-choline 12 was indeed able to solubilise SteD_ala13_. However, SteD_ala9_, which the reviewer agrees is likely to be cytoplasmic, was also partly extracted by fos-choline 12 (Fig R1A). Mutants with corresponding phenotypes delivered during *Salmonella* infection, SteD_L43A,M44A_ and SteD_S68A,G69A_, were both clearly extracted by fos-choline 12 (Fig R1B). Therefore, in these experiments, fos-choline 12 did not differentiate between integral membrane proteins and cytoplasmic aggregates and in the absence of an appropriate “intramembrane aggregate” control it is difficult to interpret anything from this experiment. Therefore, we do not think that its inclusion adds to the manuscript. On the other hand, RIPA buffer clearly differentiated between wt SteD, which was RIPA soluble, and both ala9 and ala13 mutants, which were not soluble in RIPA buffer.
 
*The authors conclude that the ala9 mutant (at the N-terminus of the TMS1) clusters in aggregates, which is likely to be correct given its co-localization with ubiquitin. Based on these results the authors hypothesize that the mutated sequence 9 mediates targeting of SteD to the membrane for which I see no evidence, however. Sequence 9 is the major contributor to the overall hydrophobicity of TMS1 (containing two leucines) and mutation to alanines simply reduces the hydrophobicity and may lead to a semi-hydrophobic sequence that can't integrate into membranes but isn't soluble either, so it aggregates. That means, the finding is highly unspecific and does not allow to draw any conclusion towards a specific function of the sequence 9 of SteD.* 

**Response**

This point is also made by referee 3 (see below) and we thank both referees for raising this possibility. With respect to both the ala 9 mutant and SteD_L42A,M43A_ (the double point mutant within the ala9 region that recapitulates the ala9 mutant phenotype [Fig 3C and D]), we agree that misfolding (assuming that some folding of SteD does normally occur) or changes in hydrophobicity could account for failure to localise at the TGN, rather than the L42 and M43 residues conferring specific targeting information. We have modified the manuscript to include this possibility from line 306.

However, the double point mutant within the ala9 region (SteD_L42A,M43A_) has less effect on the hydrophobicity of SteD and fully recapitulates the full ala9 mutant phenotype. In addition, ala substitutions in both SteD_ala9_ and SteD_L42A,M43A_ do not affect the *in silico* predictions of TM regions by TMHMM (PMID: 11152613). It is striking that, apart from the ala13 region, no other ala substitutions in SteD, including to hydrophobic TM regions, lead to its aggregation. The ala13 mutant still localises at the TGN, so mutation of region 9 (specifically of the two residues L42 and M43) is the only modification that prevents TGN localisation. It is also noteworthy that SteD_L42A,M43A_ is competent for translocation across the bacterial envelope and vacuole membrane without aggregating.

Since interference of protein integration into membranes results in aggregation and degradation of transmembrane proteins (PMID: 27284044, 28104892), we feel that the simplest explanation is that aggregation is likely to be a direct consequence of a lack of targeting or integration and mutants that prevent integration form aggregates.

*The authors conclude that the ala13 mutant is unable to integrate into membranes. A brief analysis of the membrane integration propensity of the ala13 mutant tells that it has an even increased hydrophobicity than the wt. This finding, together with the microscopy analysis the*

*authors show throughout the paper, suggests to me that membrane integration of the ala13 mutant is very well possible but that it likely forms intramembrane aggregates. Consequently, I do not buy the main conclusion the authors draw from their findings presented in Figures 1 through 3.*

**Response**

We thank the referee for pointing out the slightly increased hydrophobicity of the ala13 mutant. There would appear to be 3 possible consequences: (1) formation of free cytoplasmic aggregates (as for the ala9 mutant), (2) formation of membrane-associated cytoplasmic aggregates (3) formation of intramembrane aggregates. The ala13 mutant is clearly membrane-associated, as seen by colocalisation with TGN46 in Fig 2A and redistribution to the ER in the presence of BFA in Fig 4A. This rules out (1). As mentioned above ‘fos-choline 12 did not differentiate between integral membrane proteins and cytoplasmic aggregates and in the absence of an appropriate “intramembrane aggregate” control it is difficult to interpret anything from this experiment’.

Proteasomal degradation occurs in the cytoplasm and misfolded membrane proteins must be first retro-translocated into the cytoplasm before they are degraded (PMID: 29719269). Blocking proteasomal degradation with MG132 would therefore recover the cytoplasmic population but should have little or no effect on the amount of protein in the membrane. SteD_ala13_ was degraded in the absence of MG132 (Fig 1B), and no protein was detectable at the TGN. This provides strong evidence that SteD_ala13_ does not integrate into the membrane. We have indicated colocalization between SteD_ala13_ and ubiquitin in the cytoplasm after MG132 addition with arrows in Fig S2A and have included the point about SteD_ala13_ being susceptible to proteasomal degradation, as evidence for its non-membrane integrated state in lines 307-309.

Furthermore, it is important to bear in mind that the SteD _S68A,G69A-HA_ mutant, which is less hydrophobic and does not affect prediction of the TM regions, recapitulates the phenotype of the ala13 mutant.

Therefore the simplest explanation of these results is that the SteD_ala13_ and SteD_S68A,G69A_ mutants are associated with the TGN membrane but fail to integrate. However, we have modified the text to take account of the formal possibility that these mutants might undergo intramembrane aggregation.

*Also the statement that the TMS of SseG suffice to localize the protein even in the absence of membrane integration is likely not correct.*

**Response**

The reviewer does not explain why they believe the conclusion is likely not correct. Both SteD_TMSseG_ and SteD_TMSseGala13_ co-localised extensively with mCherry-SseG with a distribution that was quite distinct from the Golgi- localised SteD (Fig 2E). But while SteD_TMSseG_ was fully solubilised by RIPA buffer (indicating that it underwent membrane integration), the majority of SteD_TMSSeGala13_ was not solubilised by RIPA buffer but was soluble in both 8 M urea and fos-choline 12 (Fig R1A). Since SteD_TMSSeGala13_ had a similar solubilisation profile to the SteD_ala13_ and SteD _S68A,G69A-HA_ mutants the most likely explanation is that, as argued for SteD_ala13_ (above), SteD_TMSSeGala13_ does not integrate into host membranes.

*In my view, the manuscript gets much better from Figure 4 on, where the authors show that membrane integration of SteD does not occur at the TGN but at the ER, which seems to be similar for the ala13 mutant but unfortunately, the authors do not provide data on the biochemical fractionation of the mutant (Fig. 4C).*

**Response**

We thank the reviewer for pointing out this oversight. In the revised paper we include biochemical fractionation data for the ala13 mutant after BFA treatment in Fig 4C.

*I suggest to limit the manuscript to the data investigating the site of SteD integration and the trafficking and to omit the uncertain membrane integration data. The manuscript would much gain from this and shed new light on the intricate integration of bacterial effectors into the host’s cell biology. Interestingly, the title of the manuscript already points in this direction.*

*As it is right now, all statements based on the current findings that relate to a role of sequence 9 in membrane targeting and of sequence 13 in membrane integration should be toned down substantially or better be removed.*

**Response**

We feel that the work on targeting and integration of SteD are crucial aspects of its functional characterisation. However, in response to these comments, we have toned down our conclusions about the exact role of both region 9 and 13 and included a caveat that it is also possible that the ala9 mutant leads to misfolding and the ala13 mutant forms aggregates in the membrane in lines 306-307.

Reviewer #3:

*My main concern is that misfolding and aggregation of the mutant SteD proteins could account for the defects in membrane localization and function rather than specific functional requirements for region9 and region13. However, I am not sure how this can be best addressed since even the mutants with only two alanine substitutions seem to be targeted for degradation.*

**Response**

See also responses to Reviewer 1 above. If SteD_ala13_ and SteD_S68A,G69A_ were to have misfolded upon expression both would have been expected to form mislocalized cytoplasmic aggregates - yet both mutants, like wild-type SteD, localised at the TGN and SteD_ala13_ responded to BFA by reversible redistribution to the ER, revealing a stable interaction with a host membrane component. Therefore, these mutants do not have a defect in membrane localisation.

With respect to SteD_ala9_ and SteD_L42A,M43A_, neither mutations affect the in silico prediction of TM regions. It is also noteworthy that SteD_L42A,M43A_ is competent for translocation across the bacterial envelope and vacuole membrane. Since interference of protein integration into membranes results in aggregation and degradation of transmembrane proteins (PMID: 27284044, 28104892), the simplest explanation is that aggregation is likely to be a direct consequence of a lack of targeting or integration and mutants that prevent integration form aggregates.

However, we accept the criticism and have modified the text from line 306 to explain our reasoning more clearly and to indicate the possibility that the ala9 region might not confer the Golgi-targeting property of SteD.

**Minor Issues:** 

Reviewer #1:

*1. My main concern is with the SteD-AP1 interaction, which does not appear very convincing given how little of AP1 is detected in the SteD immunoprecipitates. While the authors use SseG as a negative control for comparison, I am wondering if the fraction of AP1 that interacts with SteD (how much of the total AP1 is it?) provides enough relevance of AP1-dependent transport of SteD from the TGN to endosomes and whether an AP1-independent pathway also operates. This may also explain why the AP1 knockdown has little effect on SteD-mediated decrease in surface MHCII. Could the authors show as comparison how much a known AP1-dependent host protein co-IP AP1? Also, does AP1 label SteD-containing TGN-derived vesicles? Arguably, the loss of colocalization between SteD and MHCII upon AP1 knockdown and the identification of the inverted di-leucine motifs in SteD supports the role of AP1 in SteD traffic, but perhaps this aspect of the study could be strengthened or further discussed.* 

**Response**

We thank the referee for these comments. The amount of AP1 that interacted with GFP-SteD might appear small, but it was specific, reproducible and statistically significant (Fig 5A and B). The interaction between adaptor proteins and cargoes is known to be transient during formation of the vesicle. After release of the vesicle, adaptors and coat proteins are rapidly disassembled (PMID: 15922462). In addition, only a small fraction of total cellular SteD is likely to interact with AP1 at any given time. Therefore, it does not seem unsurprising to us that a relatively small amount of AP1 was detected in the SteD immunoprecipitates.

With respect to the suggestion to use a known AP-1 dependent host protein as a comparison, we do not believe this would be informative since (a) the amount of AP1 pulled down by different proteins with different antibodies would not be comparable (b) the amount of AP1 interacting with different cargos is likely to be different and (c) it would not alter interpretation of the reproducible and significant association between AP1 and SteD.

With respect to the question ‘*does AP1 label SteD-containing TGN-derived vesicles’* we have labelled AP1 in cells expressing GFP-SteD or in cells in which transferrin receptor, an endogenous AP1 cargo is also labelled. We detect only a small amount of colocalisation in vesicles for both examples (Fig R2, indicated with arrows). This is comparable to previous published examples of TFR colocalisation with AP1 (PMID: 31895934).

We do not think that these results add much to the very clear immunoprecipitation data. However, we would be happy to include these results if the Editor/Reviewer feels they are necessary.

*Arguably, the loss of colocalization between SteD and MHCII upon AP1 knockdown and the identification of the inverted di-leucine motifs in SteD supports the role of AP1 in SteD traffic, but perhaps this aspect of the study could be strengthened or further discussed.* 

It is possible that there are additional pathways for SteD traffic, and it is also possible that alternative trafficking pathways can compensate for the knockdown of AP1 (PMID: 22902756). The discussion already includes possible explanations for the lack of effect on MHCII surface levels after AP1 knockdown. However, as the reviewer has pointed out the role of AP1 in SteD traffic is supported by AP1 knockdown and identification of a non-interacting mutant of SteD.

*2. Fig. 1B: the expression levels of SteDala9 and SteDala13 still remain very low upon MG132 treatment. Could this be due to a lack of solubility of SteDala9 and SteDala13 aggregates? How do the authors envision that SteDala13 traffics to Golgi membranes if it is in an insoluble state?*

**Response**

In Fig 1B the cells are lysed in Laemmli buffer with a final concentration of 2 % SDS. This is the same buffer that is used to solubilise aggregates following biochemical fractionation so both mutants are soluble in Laemmli buffer. The GFP fluorescence levels of SteD_ala9_ and SteD_ala13_ are also lower than the wt as measured by flow cytometry (Fig S1B), therefore the difference in protein levels is not due to lack of solubility.

Both wt SteD and SteD_ala13_ traffic to Golgi membranes, potentially through interaction with a host ER/Golgi membrane component. When membrane integration is disrupted, hydrophobic transmembrane domains are degraded in the cytoplasm so it is likely that SteD_ala13_ only aggregates and is degraded when it has failed to integrate. We have changed the discussion (lines 312-315) to read that following targeting to the Golgi and lack of integration “the prolonged presence of the exposed hydrophobic transmembrane domains in the cytoplasm causes aggregation.”

*3. Line 235: should be MHCII, not MCHII*

**Response**

Corrected. Many thanks.

*4. Fig. S1B: the legend of the flow plot should indicate MHCII, not MHII*

**Response**

Corrected. Many thanks.

Reviewer #2:

*I miss information on how the used plasmids were created. Plasmid sequences as well as primers and molecular biology operations that would help to re-create them are missing. As it is, it is not possible to know which sequence of SseG was exactly used for the experiments shown in Figure 3 and how the used fusions look like exactly.*

**Response**

We apologise for this oversight. Please find a list of primers used in Table S4 and a detailed explanation of plasmid construction in the methods section from line 368.

Reviewer #3:

*Figure 4D. The way surface expression of MHCII is shown here is confusing compared to Fig S1C where the geometric mean is shown. Why not show it the same way in both figures?*

**Response**

The reason we use percentage mMHCII surface level in Fig 4D and also 1C, 3B and 7E is to provide an internal control for labelling. This information has been added to the methods in lines 424-426. In Fig S1C the surface levels are used to check for an MG132 effect on steady-state mMHCII surface levels. This wouldn’t be picked up by comparing GFP positive with GFP negative cells. An explanation for this has been added to the figure legend.
